# A Systematic Review and Meta-Analysis of Serum Adiponectin Measurements in the Framework of Dog Obesity

**DOI:** 10.3390/ani10091650

**Published:** 2020-09-14

**Authors:** Alberto Muñoz-Prieto, José Joaquín Cerón, Silvia Martínez-Subiela, Vladimir Mrljak, Asta Tvarijonaviciute

**Affiliations:** 1 Clinic for Internal Diseases, Faculty of Veterinary Medicine, University of Zagreb, Heinzelova 55, 10000 Zagreb, Croatia; alberto.munoz@um.es (A.M.-P.); vmrljak@vef.hr (V.M.); 2 Interlab-UMU, Regional Campus of International Excellence “Mare Nostrum”, University of Murcia, 30100 Murcia, Spain; jjceron@um.es (J.J.C.); silviams@um.es (S.M.-S.)

**Keywords:** adiponectin, obesity, serum, dogs, meta-analysis

## Abstract

**Simple Summary:**

Adiponectin is a molecule with biological activity that is closely linked with obesity and obesity-related problems. Despite some literature publications, there is a lack of consensus about how adiponectin changes in canine obesity. Therefore, a meta-analysis is performed here to assess the global performance of adiponectin concentrations in terms of obesity in dogs. After considering 20 different studies, including a total sample size of 366 dogs with obesity and 349 normal weight dogs, the meta-analysis indicates that adiponectin in serum is lower in obese dogs compared with normal weight dogs and increases after weight loss programs.

**Abstract:**

Adiponectin is an abundant plasma protein that is closely related to obesity and obesity-related pathologies. The molecule can be found in three different isoforms, each with different biological activities. Studies on canine obesity have suggested that adiponectin concentrations are decreased in obesity; however, no canine meta-analyses have been performed that feature all the required data. The aim of this study is to perform a systematic review and meta-analysis of studies that pertain to total and high molecular weight (HMW) adiponectin in relation to canine obesity. From 20 different studies, a total of 366 dogs with obesity and 349 normal weight dogs are included in the meta-analysis. Client-owned dogs were most represented, accounting for 54.3% of the dogs used, while experimental dogs enrolled in the studies made up the remaining 45.7%. The concentrations of total adiponectin in dogs with obesity were significantly lower compared with normal weight dogs. Additionally, adiponectin concentrations were significantly higher in dogs after a successful weight loss protocol compared to the start of the protocol and were significantly lower in dogs after gaining weight. In conclusion, although caution should be taken due to the relatively low number of studies that exist and the high heterogeneity between them, this meta-analysis indicates that adiponectin is decreased in obese dogs.

## 1. Introduction

Adiponectin is an adipokine that is mainly secreted by adipose tissue and also in minor quantities by other tissues, such as salivary glands [1], bone cells [2], and cardiomyocytes [3]. It is one of the most abundant plasma proteins, accounting for approximately 0.01% to 0.05% of total plasma proteins [4,5], and exerts effects on different tissues, including fat, the liver, skeletal muscle, bones, cardiac muscle, and the endothelium via widely distributed receptors [2,6]. The adiponectin structure is based on a monomeric 30 kDa protein that is capable of post-translational modification into different multimers [7]. In the blood stream, adiponectin can be found in three different isoforms, namely, low molecular weight (LMW) forms integrated by a 90 kDa trimer, middle molecular weight (MMW) forms that are composed of two trimers, and high molecular weight (HMW) forms consisting of noncovalent aggregates of six or more trimers [8]. In humans, the HMW form is considered to be the most bioactive form of adiponectin [9,10]. 

Adiponectin is an insulin-sensitizing and anti-inflammatory molecule that is intensively studied in the context of obesity and obesity-related pathologies [11]. In humans and experimental animals such as mice and rats, it is well documented that adiponectin concentrations are decreased when these conditions are present [3,12]. Despite the importance of obesity and the involvement of adiponectin in its pathophysiology, there are no meta-analysis studies of canine obesity to date, unlike the human species [13,14]. The objective of this study is to perform a systematic review and meta-analysis in order to critically assess studies of both total and HMW adiponectin in the context of canine obesity.

## 2. Materials and Methods

The preferred reporting items for systematic reviews and meta-analysis (PRISMA) statement guidelines [15] were followed when performing this study.

### 2.1. Search Strategy

**Figure 1:**
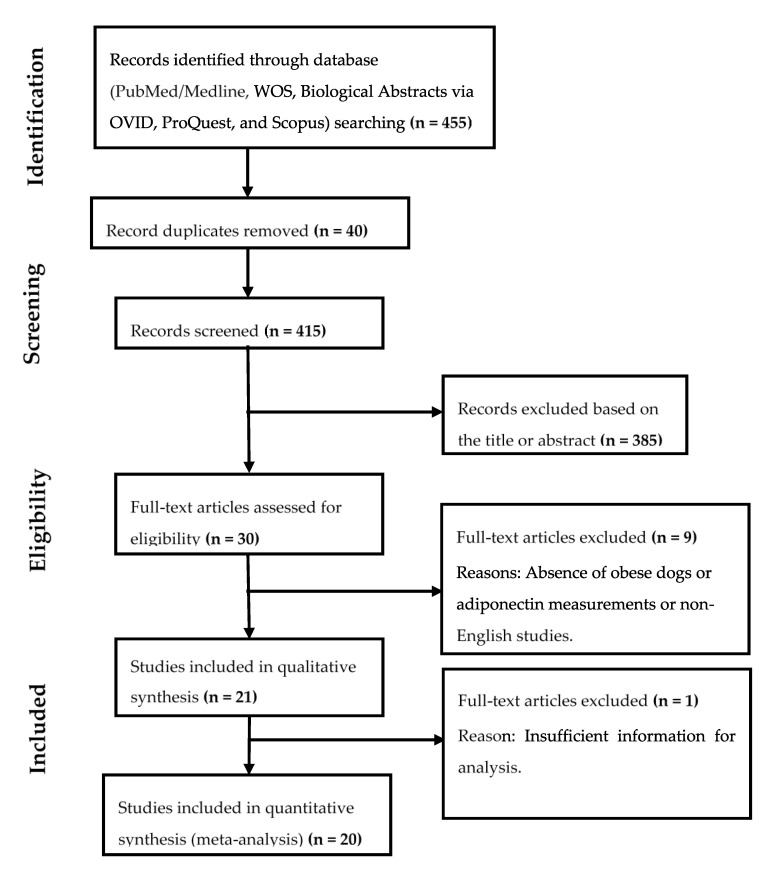
Flow diagram of literature selection for the meta-analysis. WOS: Web of Science.

We searched the PubMed, Web of Science (WOS), Biological Abstracts via OVID, ProQuest, and Scopus databases for peer-reviewed studies reporting on serum adiponectin concentrations in overweight or obese dogs from 2000 to 2019 (i.e., until December of 2019). The keyword terms were “adiponectin”, “concentration”, “levels”, “serum”, “obesity”, and “dogs”, and the index terms were the following: “Serum adiponectin in canine obesity”, or “plasma adiponectin in canine obesity”, or “serum adiponectin in obese dogs”, or “plasma adiponectin in obese dogs”. Specifically, for studies that determine some adiponectin isomers, the keywords “isomers” and “HMW adiponectin” were added while searching. All searches were limited to original articles. The full search strategies in the electronic databases are shown in Table S1. All available studies were independently reviewed by two investigators (AMP and AT). There were no disagreements between the two investigators regarding the inclusion or exclusion of articles. Figure 1 shows a flow diagram that summarizes all stages of the systematic review process, including the numbers of studies identified at each stage and any reasons for exclusion.

### 2.2. Inclusion Criteria

The retrieved studies were screened based on the titles, abstracts, and contents. Studies were identified as eligible for inclusion if they met the following criteria: (1) Observational studies (case–control) or experimental (clinical trial) studies; (2) studies reporting circulating adiponectin concentrations in obese patients versus controls; (3) studies reporting circulating adiponectin concentrations in dogs submitted to a weight loss or weight gain program; (4) peer-reviewed studies. Excluded studies are described in Table S2. The exclusion criteria were the following: Absence of obese dogs in the study; absence of reporting adiponectin measurements; and papers in which dogs were under treatment. Additionally, articles that were not written in English were removed.

Studies using more than one experimental design or more than one assay method for serum adiponectin determination were included. Some studies showed more than two groups for the comparative analysis of serum adiponectin (i.e., normal weight, overweight, and obese dogs); however, for this meta-analysis, the extremes (obese versus normal weight dogs) were selected in order to reflect adiponectin differences in clearly established canine obesity. In the specific case of Bae and Oh (2019) [16], only the group of dogs with most significant body condition score (BCS) reduction was included (group B).

### 2.3. Coding Data

Data extraction was performed for the studies found by a standardized Excel spreadsheet, that was used to record all the relevant data and variables to be analyzed (Table S3). The data extracted were the following: Publication year, study title, first author, study country, study design, sample size, assay method used, assay validation (yes or no), gender, breed, number of groups used in the study and description, BCS, and values of the mean (SD) or median (range) of total adiponectin. For studies reporting outcomes as medians and ranges, the means and SDs were calculated using the methods described previously [17].

### 2.4. Risk of Bias and Quality Assessment

Studies included in the qualitative and quantitative analyses were assessed independently for the risk of bias by two authors (A.M.-P. and A.T.) using the guidelines included in the Cochrane Handbook for Systematic Review of Interventions [18]. The following sources of bias were assessed: Random sequence generation, allocation concealment, blinding of participants and personnel, blinding of outcome assessment, incomplete outcome data, selective reporting, and other sources of bias. Each domain was given a quality score of either a high or low risk of bias. If the data were insufficient to make a reasonable judgment, the domain was classified as an unclear risk of bias. Another author (J.J.C.) was consulted when discrepancies were found in the comparison of the two independent assessments.

Only peer-reviewed and articles in English language were considered for inclusion in this meta-analysis. This fact may under-represent the total literature available, which may introduce a publication bias. The recruited studies included all the information required and met the inclusion criteria. Additionally, they all clearly showed mean or median values in the text, or these values were represented graphically, clearly describing their subject recruitment criteria, sampling materials, and methods.

To evaluate the robustness of our findings, a sensitivity analysis was conducted by repeating the meta-analyses and excluding one study at a time.

### 2.5. Statistical Analysis

The RevMan software package (version 5 for Mac OS, Cochrane Review Manager, London, UK) was used for the statistical procedures of meta-analysis. The means and standard deviations of adiponectin concentration were used for the meta-analyses and the standardized mean difference (SMD) was chosen as the effect measure, along with a 95% confidence interval (CI). The SMD was selected due to the different analytical conditions of the tests with different units used for adiponectin determination as previously reported [19]. The inverse variance method was used here in order to determine weighted mean differences for continuous data. The study heterogeneity was reported as I^2^, which describes the percentage of variability in the estimated effect owing to heterogeneity rather than chance. Here, 30% to 60% shows moderate heterogeneity, while percentages greater than 60% represent substantial heterogeneity [20]. Next, the random effect model (REM) was applied. Visual inspection of asymmetry in funnel plots was conducted to detect publication bias. 

The outcome of interest selected in the analysis was the difference in serum or plasma adiponectin concentrations between obese and normal weight dogs. The analysis was divided into subgroups according to the design of the study, i.e., observational (case–control studies) and experimental (clinical trials where dogs were submitted to a weight loss or weight gain protocol) subgroups. In addition, serum adiponectin concentrations were compared between obese and normal weight dogs in terms of being experimental or client-owned dogs.

## 3. Results

### 3.1. Literature Search and Study Selection

A total of 455 published articles were considered after a comprehensive literature search of the PubMed/Medline, WOS, Biological Abstract via OVID, ProQuest, and Scopus databases (Figure 1). After the removal of duplicates and non-relevant studies, based on titles and abstracts, 30 full-text articles were initially selected for inclusion into the meta-analysis. From these 30 articles, 10 were excluded (Table S2). Nine articles were excluded from qualitative analysis because they did not fully match the inclusion criteria (i.e., they had no comparative data between obese and normal weight dogs) or were not written in English. One article was excluded because sufficient information for meta-analysis was not available since SD values could not be obtained from the data reported. For that article, only narrative results are presented in our review. Finally, 20 studies were included in the meta-analysis for total adiponectin determinations, and within them, three that also reported levels of HMW isomers were used for further comparison. Descriptions of the studies included in the meta-analysis for total and HMW adiponectin may be found in Table 1 and Table 2, respectively. In addition, descriptive information about the data of the mean adiponectin concentrations and BCS groupings for obese and normal weight dogs is presented in Table 3.

A total of 366 dogs with obesity and 349 dogs with a normal weight from 20 different studies in which circulating serum or plasma adiponectin concentrations were reported, were compared in the meta-analysis. Client-owned dogs were most represented, accounting for 54.3% of the dogs used, while the experimental dogs enrolled in the studies represented 45.7%. The sample sizes ranged from 5 [21] to 76 [22] dogs. There were 13 case–control and 9 experimental (clinical trial) studies. Relative to the clinical trials, five studies featured a weight loss protocol [16,21,23,24,25] and dogs from four studies featured a weight gain protocol [26,27,28,29]. One study [26] included both designs with different dog populations (case–control and clinical trial) and one study used two different methods to determine serum adiponectin levels [30]. In one study [31], the mean concentrations of total and HMW adiponectin were obtained by elucidating the mean value from the figure in the manuscript and then the SD was found via calculation of the standard error using the 95% CI and sample size [17]. Finally, for one article [32], only narrative results will be presented because the SD values of adiponectin concentrations were not available. In this work, adiponectin was measured in dogs after body weight reduction and the results were expressed in a log-transformed graph.

### 3.2. Risk of Bias and Quality Assessment

**Figure 2:**
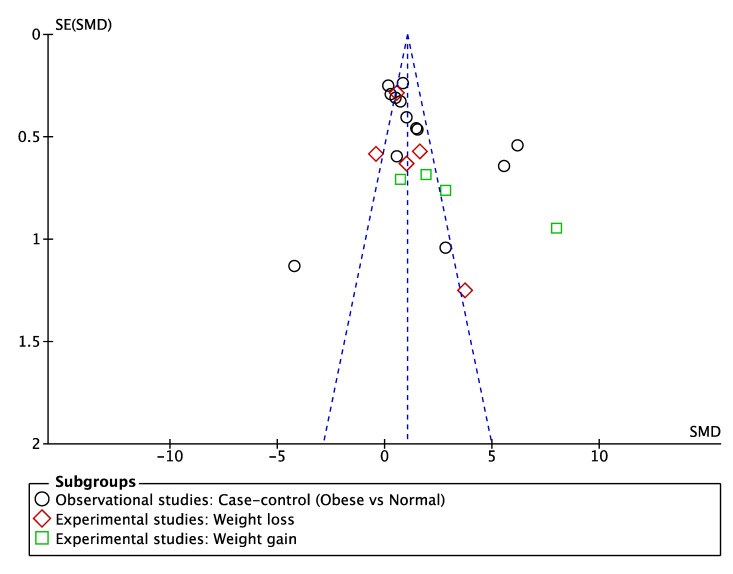
Funnel plot of meta-analysis with a 95% CI.

Owing to the participants being dogs, blinding of the studies was assumed; hence, the study was a assigned a low bias risk in this category. Similarly, a low risk rating was attributed to the blinding of the outcome assessment because the main outcome (adiponectin concentrations) was represented by a numeric value obtained from laboratory tests. The authors had no conflicts of interest to declare in relationship to their studies. Consequently, the selection, attrition, reporting, and other possible biases were the main indicators of the validity of the included studies. The risk of bias assessment is reported in Table 4. The funnel plot of the standard error of SMD against SMD for adiponectin determinations was symmetric and did not suggest a publication bias (Figure 2).

### 3.3. Sensitivity Analysis

The sensitivity study showed that when all data were considered, removing one study at a time did not alter the overall results, indicating a minor effect of individual studies on the pooled estimation and result consistency; however, within the weight loss subgroup analysis, the removal of one study [25] resulted in a loss of significance of the overall size effect. In addition, in the weight gain subgroup analysis, the subtraction of the study of Adolphe [28] or Park [29] reduced the overall effect to be below significance. 

### 3.4. Circulating Total Adiponectin Concentrations in Obese versus Normal Weight Dogs

**Figure 3:**
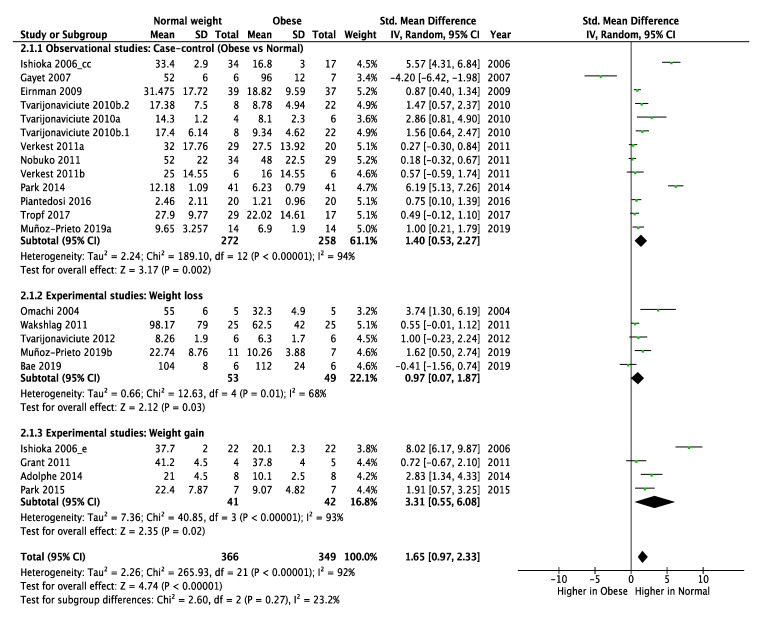
Subgroup (observational studies, i.e., case–control, and experimental studies, i.e., weight loss and weight gain) meta-analysis findings of circulating adiponectin concentrations in obese versus normal weight dogs. SD: Standard deviation; Std: Standardized; IV: Interval variable; CI: Confidence interval.

Overall, the concentrations of adiponectin in dogs with obesity were significantly lower compared with normal weight dogs (SMD = 1.65, 95% CI: 0.97–2.33, *p* < 0.001) (Figure 3).

The analysis of the subgroup of case–control studies represented 61.1% of dogs and adiponectin concentrations were significantly lower in obese dogs compared with normal weight dogs (SMD = 1.40, 95% CI: 0.53–2.27, *p* = 0.002), being the subgroup in which more differences were seen between adiponectin values in obese dogs versus normal weight dogs. 

In the case of clinical trials included in the meta-analysis, five of them were weight loss-based studies (22.1% of dogs) [16,22,24,25,26]. The adiponectin concentrations were significantly higher in dogs after a successful weight loss protocol compared to the start of the protocol (SMD = 0.97, 95% CI: 0.07–1.87, *p* = 0.03). 

Four clinical trial studies determined adiponectin concentrations in dogs during obesity development (16.8%) [27,28,29,30], and in this case the circulating adiponectin concentrations were significantly reduced when dogs became obese when compared to the initial condition (SMD = 3.31, 95% CI: 0.55–6.08, *p* = 0.020).

### 3.5. Circulating HMW Adiponectin Concentrations in Obese vs. Normal Weight Dogs

**Figure 4:**
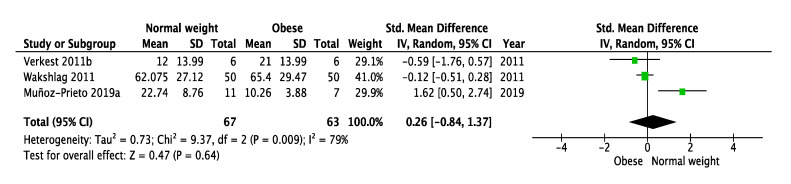
Meta-analysis of circulating HMW adiponectin concentrations in dogs after a weight loss program. SD: Standard deviation; Std: Standardized; IV: Interval variable; CI: Confidence interval.

Three studies reported HMW adiponectin concentrations comparatively between obese versus normal weight dogs [23,24,31]. The variations in HMW isomers showed no significant trend to be higher in normal weight dogs compared with obese dogs among the three studies involved (SMD = 0.26, 95% CI: −0.84–1.37, *p* = 0.64) (Figure 4).

### 3.6. Effect of Type of Experimental Subject (Experimental or Client-Owned Dog) in the Determination of Circulating ADP Concentrations in Obese Dogs Compared with Normal Weight Dogs

**Figure 5:**
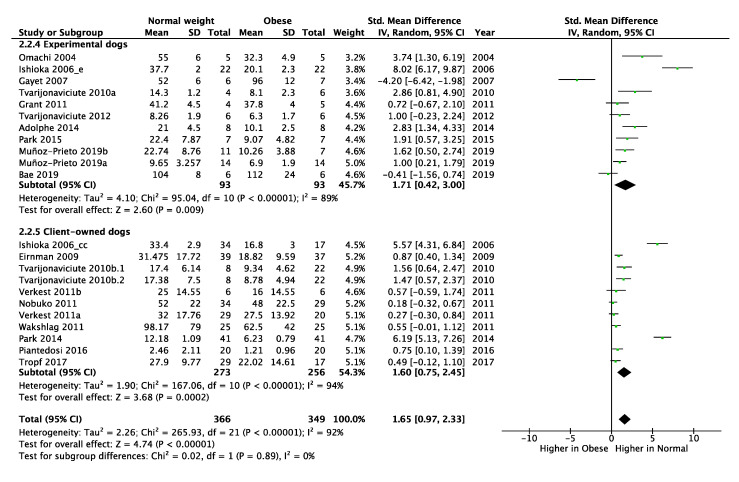
Subgroup (experimental and client-owned dogs) meta-analysis of circulating total adiponectin concentrations in obese versus normal weight dogs. SD: Standard deviation; Std: Standardized; IV: Interval variable; CI: Confidence interval.

The analysis of changes in total adiponectin concentrations between obese and normal weight dogs, in terms of the use of experimental (animals bred and sold for experimental use only) or client-owned dogs, showed variations of higher significance when adiponectin was measured in client-owned dogs (*p* < 0.001) compared with experimental dogs (*p* = 0.009) (Figure 5). 

## 4. Discussion

Obesity is currently the most commonly occurring metabolic disease in dogs, causing severe comorbidities and thus decreasing their quality of life and lifespan [33,34,35]. Therefore, understanding the relevant pathophysiological mechanisms is of crucial importance in order to combat this disease. Adiponectin is one of the key hormones in the development of obesity-related pathologies [36], and thus understanding its behavior in canine obesity could contribute to better understanding this disease in this species.

The results of the present meta-analysis revealed a significant reduction of adiponectin concentrations in dogs with obesity. This is a well-proven association in human studies, where obese patients have reduced circulating blood adiponectin values when compared to normal weight people [37,38], and there is also an inverse correlation between adiponectin concentration and obesity or visceral adiposity that normalizes following weight loss in humans [39]. Research in human and rodent models has consistently demonstrated the role of adiponectin as an important physiological regulator of obesity-related alterations [12]. In the case of the studies involving dogs with obesity that have been analyzed in this meta-analysis, the same trend was found. Subgroup analysis showed that in case–control studies comparing obese and lean animals, and also in experimental (clinical trial) studies, when the dogs were analyzed after increasing or reducing their weight, adiponectin concentrations were lower in obese dogs. We are aware that there are scarce numbers of studies available that address the behavior of adiponectin in canine obesity; however, in most of the studies, the adiponectin behavior is similar to that described in human beings, where lower serum adiponectin concentrations are detected in patients with obesity and these levels raise after weight loss [38,39]. This trend was also revealed in another work studying canine obesity, comparing obese versus normal weight dogs [32]. This article was not included in the present meta-analysis due to the lack of available quantitative results. The similarities revealed for both the human and canine species allow the assumption of canines as a possible model for the study of obesity in humans.

Adiponectin isomer distribution is of high importance in terms of the biological activity of adiponectin [40]. In human medicine, the HMW form is the most related with metabolic function [10], and in our study we focused on the analysis of this isomer. Three studies matching our inclusion criteria that measured HMW isomers in dogs with obesity were identified. However, only one [24] found statistically significantly increased HMW isoforms in lean dogs compared with normal weight dogs. The high heterogeneity of these studies, principally in terms of the methodologies used for HMW adiponectin quantification, could explain the different results reported. For instance, in the study performed by Muñoz-Prieto et al. [24], concentrations of HMW adiponectin were determined using a specific protease digestion protocol followed by an ELISA determination, while in the study performed by Verkest et al. [31], a semi-quantitative method based on Western blotting analysis was employed. However, other factors such as the study design, animal populations (experimental or client-owned), and demographic characteristics of the dogs (different ages, sexes, or breeds), among other factors, could have affected the results. Therefore, further large-scale studies evaluating the behavior of adiponectin isoforms in the context of canine obesity are expected to achieve a better understanding of the physiology of adiponectin.

The analysis of the effect of different dog populations in terms of adiponectin determination showed no differences when using either experimental or client-owned dogs. We are aware that there could be a possible influence of dog characteristics regarding laboratory determination, because using experimental animals can create homogeneous populations with environmentally controlled conditions, thus limiting confounding factors for result interpretations. Using client-owned dogs leads to greater exposure to uncontrolled variable factors but better extrapolation with the actual canine population. However, the importance in the meta-analysis of both groups was distributed equally and we did not see any differences here. 

The sensitivity analysis of adiponectin indicated that these conclusions when obese versus normal weight dogs were compared are robust, since the removal of individual studies resulted in insignificant pooled effects. However, weight loss and weight gain subgroup analysis could be affected by the removal of individual studies and therefore results should be taken with caution in these situations. 

The main limitations of the present study are the relatively low number of studies considered and the high heterogeneity between them. This stems from the inclusion of different dog populations (client-owned and experimental dogs) with different environments, a variety of dog breeds with different genetic backgrounds, demographic differences or reproductive statuses, different analytic methods for adiponectin determinations in serum or plasma, and different clinicopathological features. Moreover, the inclusion of only peer-reviewed publications written in English may under-represent the total literature available, which may introduce a publication bias. These factors may have a substantial effect on the results. Although we did not conduct subgroup analyses for sex or reproductive status due to the lack of individual data for dog adiponectin concentrations being reported in the published articles regarding these factors, previously reported data indicate no influence of these parameters in terms of adiponectin concentrations in dogs [41]. Other variables of interest within the scope of this meta-analysis (sex distribution, reproductive status, and BCS) were not always available. This could be relevant to the results and should be considered in the design of future studies.

## 5. Conclusions

In conclusion, although caution should be taken due to the relatively low number of studies that exist and the high heterogeneity between them, this meta-analysis provides insight into the study of adiponectin in a canine obesity framework, revealing that adiponectin is decreased in obese dogs.

## Figures and Tables

**Table 1:** Study characteristics of studies determining serum or plasma total adiponectin concentrations included in the meta-analysis.

No.	Study	Country	Assay Method	Study Design	Methodology	N	Dog Condition	Gender (M/F)	BCS Mean or Median		
									Point Scale	Obese	Normal Weight
1	Ishioka et al. (2006)_cc	Japan	ELISA ^1^	Case-control		71	Client-owned	-	5	5	3
	Ishioka et al. (2006)_e	Japan		Clinical trial	Weight gain	44	Experimental	12/22	-	-	-
2	Gayet et al. (2007)	France	RIA ^2^	Case-control		13	Experimental	0/13	-	-	-
3	Omachi et al. (2007)	Japan	ELISA ^1^	Clinical trial	Weight loss	5	Experimental	-	5	4–5	-
4	Eirman et al. (2009)	United States	Luminex-based ^3^	Case-control		76	Client-Owned	38/38	9	8	5
5	Tvarjionaviciute et al. (2010)a	Spain	ELISA ^4^	Case-control		15	Experimental	8/7	5	5	3
6	Tvarijonaviciute et al. (2010)b.1	Spain	ELISA ^4^	Case-control		30	Client-owned	14/16	5	>3	3
	Tvarijonaviciute et al. (2010)b.2	Spain	ELISA ^5^	Case-control		30					
7	Grant et al. (2011)	United States	ELISA ^5^	Clinical trial	Weight gain	9	Experimental	0/9	9	8.1	4.2
8	Wakshlag et al. (2011)	United States	Luminex-based ^3^	Clinical trial	Weight loss	50	Client-owned	-	9	8	5
9	Verkest et al. (2011)a	Australia	ELISA ^1^	Case-control		49	Client-owned	-	9	8.5	4.5
10	Verkest et al. (2011)b	Australia	ELISA ^1^	Case-control		12	Client-owned	6/6	9	8.5	4.5
11	Nobuko et al., 2011	Japan	ElISA ^1^	Case-control		63	Client-owned	33/30	5	>4	3
12	Tvarijonaviciute et al. (2012)	Spain	ELISA ^4^	Clinical trial	Weight loss	6	Experimental	0/6	5	5	3
13	Adolphe et al. (2014)	Canada	ELISA ^5^	Clinical trial	Weight gain	8	Experimental	3/5	9	-	-
14	Park et al. (2014)	South Korea	ELISA ^5^	Case-control		82	Client-owned	32/50	9	8.09	4.32
15	Park et al. (2015)	South Korea	ELISA ^5^	Clinical trial	Weight gain	14	Experimental	-	9	8.71	5
16	Piantedosi et al. (2016)	Italy	ELISA ^5^	Case-control		40	Client-owned	10/30	9	7.4	5
17	Tropf et al. (2017)	United States	ELISA ^1^	Case-control		46	Client-owned	19/27	9	8	4
18	Bae and Oh (2019)	South Korea	ELISA ^1^	Clinical trial	Weight loss	6	Experimental	5/1	9	6.4	4.7
19	Muñoz-Prieto et al. (2019)a	Spain	ELISA ^4^	Clinical trial	Weight loss	14	Experimental	14/0	5	4.7	4
20	Muñoz-Prieto et al. (2019)b	Spain	AlphaLISA ^6^	Case-control		26	Experimental	26/0	5	5	3.2

^1^ ELISA kit (Otsuka, Tokyo, Japan); ^2^ Murine radioimmunoassay (LINCO); ^3^ Luminex-based adipokine assay (MilliplexTM MAP canine adipokine kit, Millipore, St. Charles, MO); ^4^ Human Adiponectin ELISA, High Sensitivity Kit, BioVendor-Labaratorni medicina; ^5^ Canine Adiponectin ELISA Kit; Millipore, St. Charles, Missouri, USA; ^6^ AlphaLISA immunoassay (Muñoz-Prieto et al., 2019). N: Sample size; M: Male; F: Female; BCS: Body condition score; cc: Case–control (observational study); e: Experimental study (clinical trial).

**Table 2:** Study characteristics of studies determining serum or plasma high molecular weight (HMW) adiponectin concentrations included in the meta-analysis.

No.	Study	Country	Assay Method	Study Design	Methodology	N	Gender (M/F)	BCS Mean or Median		
								Point Scale	Obese	Normal Weight
1	Wakshlag et al. (2011)	United States	ELISA ^1^	Clinical trial	Weight loss	50	-	9	8	5
2	Verkest et al. (2011)b	Australia	ELISA ^2^	Case–control		12	6/6	9	8.5	4.5
3	Muñoz-Prieto et al. (2019)a	Spain	AlphaLISA ^3^	Clinical trial	Weight loss	26	26/0	5	5	3.2

^1^ HMW Adiponectin ELISA Kit; Millipore, Concord, MA, USA; ^2^ ELISA kit (Otsuka, Tokyo, Japan); ^3^ AlphaLISA immunoassay (Muñoz-Prieto et al., 2019).

**Table 3:** Body condition score and adiponectin concentrations of individual studies included in the meta-analysis.

Study	Dog Condition	N	BCS	Adiponectin Concentrations
Ishioka et al. (2006)_cc	Normal weight	34	3/5	33.4 ± 2.9
	Obese	17	5/5	16.8 ± 3
Ishioka et al. (2006)_e	Normal weight	22	-	37.7± 2
	Obese	22	-	20.1 ± 2.3
Gayet et al. (2007)	Normal weight	6	-	52 ± 6
	Obese	7	-	94 ± 12
Omachi et al. (2007)	Normal weight	5	-	55 ± 6
	Obese	5	4–5/5	32.3 ± 4.9
Eirman et al. (2007)	Normal weight	39	5/9	31.4 ± 17.7
	Obese	37	8/9	18.8 ± 9.5
Tvarjionaviciute et al. (2010)a	Normal weight	4	3/5	14.3 ± 1.2
	Obese	6	5/5	8.1 ± 2.3
Tvarjionaviciute et al. (2010)b.1	Normal weight	8	3/5	17.4 ± 6.4
	Obese	22	>3/5	9.3 ± 4.6
Tvarjionaviciute et al. (2010)b.2	Normal weight	8	3/5	17.3 ± 7.5
	Obese	22	>3/5	8.7 ± 4.9
Nobuko et al. (2011)	Normal weight	34	3/5	52 ± 22
	Obese	29	>4/5	48 ± 22.5
Grant et al. (2011)	Normal weight	4	4.2/9	41.2 ± 4.5
	Obese	5	8.1/9	37.8 ± 4
Wakshlag et al. (2011)	Normal weight	25	5/9	98.1 ± 79
	Obese	25	8/9	62.5 ± 42
Verkest et al. (2011)a	Normal weight	29	4.5/9	32 ± 17.7
	Obese	20	8.5/9	27.5 ± 13.9
Verkest et al. (2011)b	Normal weight	6	4.5/9	25 ± 14.5
	Obese	6	8.5/9	16 ± 14.5
Tvarijonaviciute et al. (2012)	Normal weight	6	3/5	25 ± 14.5
	Obese	6	5/5	16 ± 14.5
Adolphe et al. (2014)	Normal weight	6	-	21 ± 4.5
	Obese	6	-	10.1 ± 2.5
Park et al. (2014)	Normal weight	41	4.3/9	12.1 ± 1
	Obese	41	8/9	6.2 ± 0.7
Park et al. (2015)	Normal weight	7	5/9	22.4 ± 7.8
	Obese	7	8.7/9	9 ± 4.8
Piantedosi et al. (2016)	Normal weight	20	5/9	2.4 ± 2.1
	Obese	20	7.4/9	1.2 ± 0.9
Tropf et al. (2017)	Normal weight	29	4/9	27.9 ± 9.7
	Obese	17	8/9	22 ± 14.6
Bae et al. (2019)	Normal weight	6	4.7/9	104 ± 8
	Obese	6	6.4/9	112 ± 24
Muñoz-Prieto et al. (2019)a	Normal weight	14	4/5	9.6 ± 3.2
	Obese	14	4.7/5	6.9 ± 1.9
Muñoz-Prieto et al. (2019)b	Normal weight	11	3.2/5	22.7 ± 8.7
	Obese	7	5/5	10.2 ± 3.8

BCS was only included in cases where authors reported it. N: Sample size.

**Table 4:** Risk of bias for the studies include in the meta-analysis.

Reference	Risk of Bias Criteria					
	Random Sequence Generation (Selection Bias)	Allocation Concealment (Selection Bias)	Blinding of Participants and Personnel (Performance Bias)	Blinding of Outcome Assessment (Detection Bias)	Incomplete Outcome Data (Attrition Bias)	Selective Reporting (Reporting Bias)
Ishioka et al. (2006)_cc	High	High	Low	Low	Low	Low
Ishioka et al. (2006)_e	Unclear	Unclear	Low	Low	Low	Low
Gayet et al. (2007)	High	High	Low	Low	Low	Low
Omachi et al. (2007)	Unclear	Unclear	Low	Low	Low	Low
Eirman et al. (2009)	High	High	Low	Low	Low	Low
Tvarjionaviciute et al. (2010)a	High	High	Low	Low	Low	Low
Tvarijonaviciute et al. (2010)b.1	High	High	Low	Low	Low	Low
Tvarijonaviciute et al. (2010)b.2	High	High	Low	Low	Low	Low
Grant et al. (2011)	Unclear	Unclear	Low	Low	Low	Low
Wakshlag et al. (2011)	Unclear	Unclear	Low	Low	Low	Low
Verkest et al. (2011)a	High	High	Low	Low	Low	Low
Verkest et al. (2011)b	High	High	Low	Low	Low	Low
Nobuko et al., 2011	High	High	Low	Low	Low	Low
Tvarijonaviciute et al. (2012)	Unclear	Unclear	Low	Low	Low	Low
Adolphe et al. (2014)	Unclear	Unclear	Low	Low	Low	Low
Park et al. (2014)	High	High	Low	Low	Low	Low
Park et al. (2015)	Unclear	Unclear	Low	Low	Low	Low
Bastien et al. (2015)	Unclear	Unclear	Low	Low	Low	Low
Piantedosi et al. (2016)	High	High	Low	Low	Low	Low
Tropf et al. (2017)	High	High	Low	Low	Low	Low
Bae and Oh (2019)	Unclear	Unclear	Low	Low	Low	Low
Muñoz-Prieto et al. (2019)a	Unclear	Unclear	Low	Low	Low	Low
Muñoz-Prieto et al. (2019)b	High	High	Low	Low	Low	Low

cc: Case–control (observational study); e: Experimental study (clinical trial).

## Supplementary Materials

The following are available online at https://www.mdpi.com/2076-2615/10/9/1650/s1, Table S1: Search strategies for PubMed, Web of Science, Scopus, ProQuest and Biological Abstracts via Ovid, Table S2: Excluded articles from database searching, Table S3: Coding data of studiess from search strategy.
